# Channeling ancestral knowledge into production: from Ecuadorian curare to a shared-benefit model that breaks Indigenous poverty

**DOI:** 10.3389/fpubh.2026.1860471

**Published:** 2026-07-21

**Authors:** Esteban Ortiz-Prado

**Affiliations:** One Health Research Group, Universidad de Las Américas, Quito, Ecuador

**Keywords:** ancestral knowledge, Ecuador, Indigenous communities, Indigenous poverty, medicine

## 1 Introduction

Indigenous ancestral knowledge is not merely cultural heritage; it constitutes proven technologies of survival developed over more than 50,000 years (1, 2). Yet history reveals a recurring pattern: when this knowledge is converted into a medically useful molecule by Western science, the originating communities are systematically excluded from the value chain. The Ecuadorian curare case is one of the clearest and least-told examples of this dynamic.

In 1938, Richard Gill, backed by financier Sayre Merrill, led an expedition into the territory of the Canelos people in the Ecuadorian Amazon (2, 3). Over 4 months he lived with Indigenous healers, documented the ritual and technical preparation of curare, and extracted 25 pounds (11.3 kg) of crude curare together with 75 botanical specimens (primarily *Chondodendron iquitamum, C. tomentosum*, and *Sciadotenia toxifera*) (2, 3). This material allowed A.R. McIntyre to perform biological standardization, A.E. Bennett to use it in convulsive shock therapy, and, in 1942, Harold Griffith and Enid Johnson to introduce it into general anesthesia (Intocostrin^®^, Squibb, New Jersey, USA) (2, 3). With this, we can observe a clear example of the direct transfer of Indigenous knowledge into pharmaceutical innovation, in which numerous actors participated. First, it was the Canelos who developed knowledge regarding the identification of the plant as well as its preparation. Richard Gill documented this knowledge during his expeditions, through interaction with Indigenous peoples, observing that it was these latter who transferred their knowledge. Subsequently, after the documentation of this knowledge, botanical classification is carried out, along with the extraction of active compounds, testing, and laboratory experiments, leading to the proper development of the pharmaceutical product. The subsequent revolution in muscular relaxation, modern surgery, and intensive care medicine would not have been possible without Canelos ancestral knowledge. Nevertheless, the communities that had safeguarded and refined this knowledge for centuries remained trapped in structural poverty. Gill himself died in 1958 disappointed that neither the Indigenous peoples nor he, as cultural bridge, had received significant commercial benefits (2, 3). The Canelos inhabit the territory south and east of Puyo, the capital of Pastaza Province, as well as the regions along the Bobonaza, Conambo, Curaray, and Villano rivers in Ecuador (4). They cultivate fiber palm, cinnamon, cacao, plantains, tobacco, and cassava, in addition to fishing and producing ceramic handicrafts. Women are primarily responsible for pottery making and gardening, with the exception of cultivating tobacco, plantains, and maize, which are generally not part of their responsibilities (4). Men, women, and children all gather wild fruits, seeds, snails, freshwater shrimp, crabs, and both terrestrial and aquatic turtles, whereas hunting in the forest is an exclusively male activity (4).

## 2 The structural problem: ancestral knowledge without economic sovereignty

This pattern repeats across the continent, examples such as the quinine, ipecac, coca, ayahuasca, and many more recent molecules. Indigenous peoples preserve the ancestral knowledge, select the plants, understand the dosages and rituals (5–7), yet when the pharmaceutical industry isolates the active compound and patents it in the United States or Europe, economic benefits flow northward (8–10). The communities are left as mere “suppliers of raw material” or, worse, are excluded entirely.

In Ecuador, a plurinational country whose 2008 Constitution explicitly recognizes Indigenous knowledge (Articles 57,402) this imbalance among original user and modern biotech adventurous is particularly painful (11). Among the rights recognized and guaranteed in Article 57 is the right to “*maintain, protect, and develop collective knowledge; their sciences, technologies, and ancestral knowledge; the genetic resources that contain biological diversity and agrobiodiversity; and their medicines and traditional medical practices.”* Furthermore, the article states that

“*all forms of appropriation of their knowledge, innovations, and practices are prohibited*.” (11)

Meanwhile, Article 402 states that

“*The granting of rights, including intellectual property rights, over derived or synthesized products obtained from collective knowledge associated with the nation's biodiversity is prohibited*.” (11)

While recognition by the Constitution represents an important step forward, this legal recognition alone is not sufficient. Intellectual property rights systems have been criticized by Indigenous peoples for promoting the commercialization and commodification of traditional knowledge under terms that are inequitable to its creators (12). Furthermore, Indigenous peoples are generally not sufficiently informed about the legal mechanisms of intellectual property, and exercising their rights and protecting their interests within these systems is often prohibitively expensive. In addition, intellectual property rights systems are largely incompatible with Indigenous cultures because they are based on the principles of individual creativity and ownership (12).

The very Amazonian and Andean communities that steward the world's greatest biodiversity also experience the highest levels of multidimensional poverty and the poorest health indicators conditions that have remained largely unchanged from the era of early explorers to that of modern drug discovery. It is necessary to implement public policies and Indigenous entrepreneurship models to bridge the gap between ancestral knowledge and economic justice. Economic justice implies that all individuals have equitable opportunities to participate in and benefit from the economy, including those who have been historically marginalized by current economic systems (13). These efforts are essential to achieving economic sovereignty, which refers to the capacity of Indigenous peoples to exercise authority over their own economic affairs, including control over taxation, trade regulation, monetary policy, resource allocation, and the right to design and implement social welfare programs according to their own priorities and governance systems (14).

## 3 The solution: linking ancestral knowledge to production

The goal is not to “protect” ancestral knowledge as a museum piece but to activate it economically under Indigenous control or co-control. Four concrete lines of action are proposed from a public health perspective:

1. **Full implementation of the Nagoya Protocol (Access and Benefit-Sharing – ABS):** Ecuador ratified the Protocol in 2014 (15). A national registry of ancestral knowledge must be created with Prior Informed Consent (PIC) and Mutually Agreed Terms (MAT) that require any researcher or company to share at least 30%−50% of the economic benefits arising from commercialization. ABS governance plays a central role in biodiversity conservation, the sustainable use of biological resources, and the protection of traditional knowledge systems (16). However, its implementation faces several important limitations. First, many of the events involving Indigenous Peoples occurred several decades before the adoption of the Convention on Biological Diversity (CBD). Consequently, current ABS frameworks have limited capacity to address historical injustices retrospectively. Furthermore, progress in ABS implementation remains uneven, with persistent shortcomings in legal frameworks, compliance mechanisms, institutional coordination, and the meaningful inclusion of Indigenous Peoples and local communities (16). In addition, several implementation issues that were left unresolved during the initial CBD negotiations continue to be debated and refined among stakeholders (17). It is also important to note that, in many cases, the principle of free, prior, and informed consent (FPIC) has not been adequately implemented. Moreover, there remains a clear lack of commitment among user countries to establish effective measures for monitoring and enforcing compliance with the ABS requirements established by provider countries (17, 18).
2. **Community-owned bioproduction enterprises:** Reduce unnecessary bureaucratic barriers and promote community-based, cooperative business models in which local discoveries can be systematically studied by regional universities and research centers. These efforts should be productively linked to private-sector partners capable of accelerating development, scaling, and commercialization. Such an ecosystem must be supported by an agile and efficient regulatory framework that facilitates innovation while ensuring equitable benefit-sharing and local participation.
3. **Collective intellectual property and collective trademarks:** Instead of individual patents, promote collective trademarks and origin certifications (“Canelos Curare – Ecuador”) that guarantee value remains within the community. Successful examples exist in Peru (“Maca Junin Pasco”) and Bolivia (“Real Quinoa”) or even Ecuador with (“Cacao Arriba”) (19–21).
4. **Intercultural public health education:** Training programs in health must include mandatory modules on sovereignty over ancestral knowledge and ethical business models. Ecuadorian universities and the Ministries of Health and Indigenous Peoples must co-design these curricula with Indigenous authorities.

Taken together, these strategies outline a structural shift from extraction to co-production, where ancestral knowledge is not merely acknowledged but actively integrated into equitable value chains. Furthermore, it is important to emphasize that these strategies should not be viewed as isolated measures operating independently. Rather, they should be approached as interdependent components of a broader transformation process. This process enables the transition from a linear model, in which ancestral knowledge is regarded merely as a source of ethnobotanical information or biological resources, while Indigenous communities remain excluded from the processes of intellectual property protection, production, and commercialization, which are instead controlled by third parties, to a circular model in which Indigenous communities are integrated into every stage of the innovation process. Within this model, governance arrangements, fair benefit-sharing mechanisms, and collective intellectual property frameworks are established to safeguard the identity and rights associated with traditional knowledge.

A review of 115 studies found that models with 100% Indigenous ownership and control were more likely to improve health and wellbeing by fostering self-determination, strengthening cultural identity, and promoting healing. These models also offered a more holistic and sustainable approach to Indigenous development (22). Similarly, evidence from Maya Indigenous communities in the Mexican state of Chiapas has shown that community enterprises serve as a mechanism for Indigenous self-determination and self-managed development. In addition, these enterprises strengthen Indigenous identity, preserve traditional culture, reinforce autonomy through self-governance, expand participation in public decision-making, and promote the protection and sustainable use of natural resources (23).

In contrast, unsuccessful experiences highlight the consequences of failing to recognize Indigenous self-determination. A clear example is Australia's *Closing the Gap* policy, which was designed to reduce socioeconomic disparities between Indigenous and non-Indigenous populations. One of the main reasons for its limited success was that it relied on a highly programmatic, government-controlled approach that failed to recognize and support Indigenous self-determination as a fundamental principle (22).

**Figure 1:**
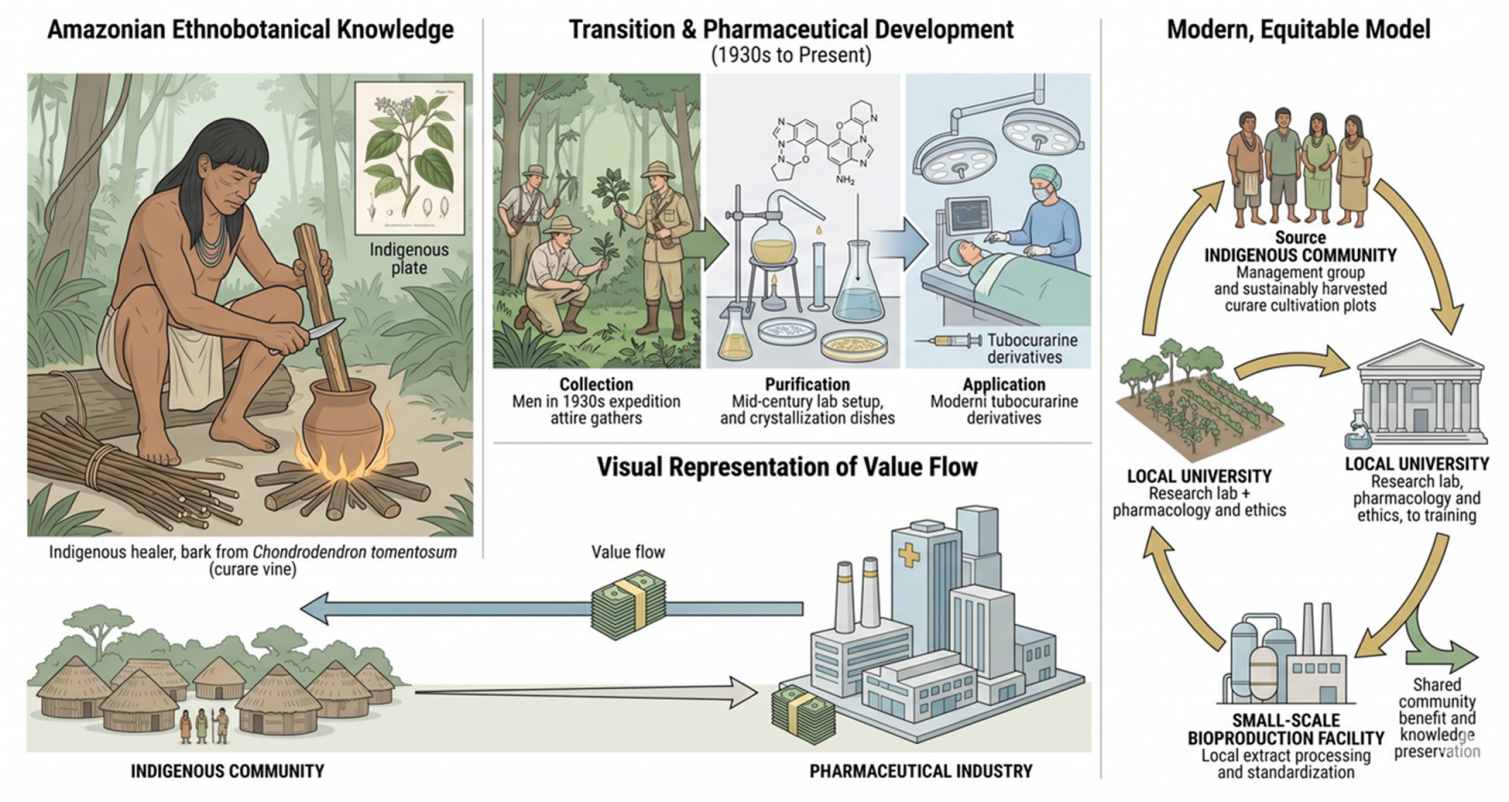
Evolution of drug development from Amazonian curare, contrasting historical extraction with a modern equitable model. From left to right: an Indigenous healer prepares curare extract from *Chondrodendron tomentosum*. Scientific expeditions and laboratory synthesis isolate tubocurarine for modern anesthesia. An overlay shows the historical unequal flow of value. The right panel proposes an equitable bioeconomy integrating Indigenous management, local university research, and small-scale bioproduction for shared benefits.

This transformation requires reimagining the entire pathway from discovery to commercialization as a shared process grounded in sovereignty, reciprocity, and justice. The conceptual framework depicted in Figure 1 illustrates this transition, contrasting the historical linear model of knowledge extraction and asymmetric benefit distribution with a circular, community-centered system in which Indigenous actors participate as co-owners of innovation and its outcomes. Only then can public health transform ancestral wisdom into sustainable material, cultural, and spiritual wellbeing. It refers to creating and maintaining a positive and inclusive environment that values inclusion and mutual benefit. It also recognizes spiritual development as a gradual process that increases an individual's obligations and responsibilities toward the community and the Earth, leading to a greater commitment to caring for the land (24, 25). True healing, grounded in the principle of “All Our Relations,” requires that the custodians of Indigenous knowledge also become the primary beneficiaries of its modern applications. This principle recognizes that all beings including humans, animals, plants, and the natural world are interconnected and interdependent. It further affirms that every person and every living being has a unique purpose, and therefore deserves respect, care, and recognition as an essential part of the interconnected web of life (26).

On the other hand, it is important to highlight the significance of Indigenous data sovereignty, which refers to the fundamental rights of Indigenous peoples to control, access, interpret, manage, and collectively own data about their communities, lands, and cultures (27). Furthermore, Indigenous knowledge systems have been recognized not merely as complementary sources of information, but as complete systems of governance, ethics, and management. Their integration with scientific knowledge offers potential solutions to global challenges in health, agriculture, and conservation, while also serving as a bridge for the integration of systematic knowledge into traditional communities for learning and development (28, 29). Finally, it is necessary to advance the decolonization of science and biodiversity conservation practices, moving toward more locally grounded, plural, socially just, and convivial forms of conservation (30).

## 4 Conclusion

Public health has an ethical duty to move from the mere “appropriation” of Indigenous knowledge to its economic co-construction. Curare was not simply a medical “discovery”; it was an act of extraction of ancestral knowledge that saved lives in the Global North while leaving its original guardians in poverty.

Today we have a historic opportunity to close that circle. Channeling ancestral knowledge into production chains controlled or co-controlled by Indigenous communities is not only reparative justice—it is the smartest and most sustainable way to advance holistic health (physical, emotional, spiritual, and economic) for Indigenous peoples. As the principle of “All Our Relations” teaches us, the healing of some cannot be built upon the poverty of others.

Only when ancestral knowledge is translated into genuine economic sovereignty can we truly say that we have honored and preserved Indigenous wisdom.
